# Implementing depression care in under-resourced communities: a school-based family resilience skill-building pilot randomized controlled trial in the United States

**DOI:** 10.3389/fpsyg.2023.1233901

**Published:** 2023-09-14

**Authors:** Lauren Marlotte, Alexandra Klomhaus, Roya Ijadi-Maghsoodi, Hilary Aralis, Patricia Lester, Kim Griffin Esperon, Sheryl Kataoka

**Affiliations:** ^1^Division of Population Behavioral Health, Jane and Terry Semel Institute for Neuroscience & Human Behavior, University of California, Los Angeles, Los Angeles, CA, United States; ^2^Department of Medicine Statistics Core, David Geffen School of Medicine, University of California, Los Angeles, Los Angeles, CA, United States; ^3^VA Health Service Research & Development (HSR&D) Center for the Study of Healthcare Innovation, Implementation & Policy (CSHIIP), VA Greater Los Angeles Healthcare System, Los Angeles, CA, United States; ^4^Department of Biostatistics, Fielding School of Public Health, University of California, Los Angeles, Los Angeles, CA, United States; ^5^Los Angeles Unified School District, Los Angeles, CA, United States

**Keywords:** adolescent, depression, mental health, family, school-based mental health

## Abstract

**Introduction:**

Youth in under-resourced communities are more likely to have greater social risk factors for mental health needs yet have less access to needed care. School-based mental health services are effective in treating common disorders such as adolescent depression; however, few have a family-centered approach, which may especially benefit specific populations.

**Methods:**

Utilizing a community-partnered approach, we adapted an established, trauma-informed, resilience skill-building family intervention for adolescents with depression. We conducted a small randomized controlled feasibility pilot of an adapted intervention in a large school district that serves predominately low-income, Latinx students in the Southwest United States between 2014-2017. Youth between the ages of 12-18 years old with a Patient Health Questionnaire (PHQ-8) score of 10 or higher, who spoke English or Spanish, were recruited from 12 school mental health clinics. Twenty-five eligible adolescents with depression and their participating caregivers were enrolled and randomly assigned to receive either the adapted intervention, Families OverComing Under Stress for Families with Adolescent Depression (FOCUS-AD), or usual care, Cognitive Behavioral Therapy (CBT) only. Most of the sample was Latinx and female. We evaluated feasibility, acceptability, and preliminary effectiveness.

**Results:**

Among participants who completed standardized assessments administered at baseline and approximately five months post-randomization (*n* = 10 FOCUS-AD, *n* = 11 CBT only), effectiveness was explored by identifying significant changes over time in adolescent mental health within the FOCUS-AD and CBT only groups and comparing the magnitude of these changes between groups. Nonparametric statistical tests were used. We found the FOCUS-AD intervention to be feasible and acceptable; participant retention was high. Adolescent symptoms of depression (measured by the PHQ-8) improved significantly from baseline to follow-up for youth in both FOCUS-AD (median decrease [MD] = 10, *p* = 0.02) and control (MD = 6, *p* = 0.01) groups, with no significant difference across the two groups. Results were similar for symptoms of PTSD (measured by the Child PTSD Symptom Scale; FOCUS-AD MD = 12.5, *p* = 0.01; CBT only MD = 7, *p* = 0.04; no significant difference between groups).

**Conclusion:**

Family-centered approaches to depression treatment among adolescents living in under-resourced communities may lead to improved mental health, although further research is warranted.

## 1. Introduction

Despite an estimated 17% of adolescents affected by depression nationally during their lifetime, few access mental health care; this is often due to systemic barriers to care, especially for youth living in structurally marginalized and under-resourced communities (Avenevoli et al., 2015; SAMHSA, 2021). Untreated adolescent depression can lead to impaired social, emotional, and cognitive development, serious health risk behaviors, and decreased school performance (Thapar et al., 2012; Vogel, 2012). Youth with depression are also at increased risk for suicide, the second leading cause of death for youth (Curtin and Heron, 2019) and this is particularly true for Latinx youth (Centers for Disease Control and Prevention, 2019). Rates of depression are higher for Latinx youth compared to non-Latinx youth (Gonzales et al., 2012; Kempfer et al., 2017). Latinx youth and youth living in under-resourced communities and in households experiencing greater social stressors are particularly vulnerable to depression (Nurius et al., 2020).

Despite potential social and structural risk factors for depression, youth living in under-resourced communities may have less access to needed mental health care than their peers. Under-resourced youth and families face significant systemic and family-level barriers to obtaining high-quality mental health care, including lack of screening for depression, poor access to local mental health services providers in convenient locations, lack of culturally-sensitive care, lack of affordable care, lack of providers, lack of insurance, cost, transportation barriers, the capability to flexibly take time off of work for appointments, and parental depression (Pumariega et al., 2005; Flores and Tomany-Korman, 2008; Chung et al., 2010; Najman et al., 2010; Keeton et al., 2012). Mufson et al. (2004) emphasize the importance of providing and researching services in school-based clinics and explain that these locations are attractive in terms of barriers presented by transportation, finances, stigma, and familiarity. Providing mental health services in schools can be one way to address some of these barriers to care in a familiar and community-based setting (Lyon et al., 2013), and may be especially important in improving access to comprehensive and coordinated care for BIPOC youth (Keeton et al., 2012). There are few researched treatments for depression with under-resourced youth and their families (Huey and Polo, 2008; Wagstaff and Polo, 2012; Pina et al., 2019), especially school-based interventions (U.S. Public Health Service, 2000), and studies are often not inclusive of BIPOC youth and families.

Stressful life events and traumatic stress can co-occur with depression (Vibhakar et al., 2019). Recent research indicates that stress has a unique relationship to depression. Causes of depression are complex; genetics combined with life and interpersonal stress may predict depression in emerging adults (Vrshek-Schallhorn et al., 2015a,b; Naviya Antony and Sultana, 2021). In cross-cultural samples, interpersonal stress may also be a significant mediator between trauma and depressive symptoms (Fung et al., 2022). Latinx populations report more stress compared to other racial/ethnic groups in the United States (American Psychological Association, 2017) and acculturative stress in Latinx adolescents is associated with greater depression symptoms (Perreira et al., 2019).

Family-centered approaches can support adolescents seeking treatment for depression (Reyes-Portillo et al., 2017), and family support is particularly important in promoting positive mental health outcomes for under-resourced youth (Vega et al., 1991). Positive family environments may decrease depressive symptoms in adolescents (Sela et al., 2020). Family-centered care has five principles: Open information sharing, respect for expertise of the family and honoring cultural differences, partnership and collaboration between families and providers, negotiation and empowerment of families, and being flexible in the care in the context of family and community (Kuo et al., 2012).

Cognitive Behavioral Therapy (CBT) is the most researched treatment for adolescent depression and has been found to be efficacious (e.g., Spirito et al., 2011). In CBT treatment for adolescent depression, families are not commonly included in treatment (Gee et al., 2020), yet family inclusion is considered optimal for depression treatment (Tursi et al., 2013). A recent meta-analysis indicated that family-based CBT, which consisted of only three studies, was superior to treatment as usual and waitlist, however, no differences were found between individual and family CBT in reducing child anxiety symptoms (Sigurvinsdóttir et al., 2020). Large-scale efficacy trials with youth at risk for depression utilizing family-oriented, strength-based, resilience-focused interventions have shown positive effects (Beardslee et al., 2003). Such interventions have promise to simultaneously increase youth engagement in and family support for needed depression services consistent with treatment. Family interventions also address broader family behavioral challenges and contextual issues that affect treatment with a strength-based paradigm and incorporate community-level stressors. Given the promising findings of involving family members in adolescent depression treatment (Reyes-Portillo et al., 2017), the unique role stress appears to play in depression, and the dearth of studies in family-based modalities to address adolescent depression in school-based clinics, we adapted a manualized evidence-based resilience skill-building family intervention called Families OverComing Under Stress (FOCUS; Lester et al., 2012) for delivery to adolescents with depression and their families in a school-based clinic setting. FOCUS is based on the theory of resilience, a positive adaptation to stress and adversity (Luthar, 2006). Lester et al. (2016) developed FOCUS from three evidence-based family-centered preventive interventions evaluated through randomized controlled trials. Components of FOCUS were informed from these interventions; one intervention for youth with parental HIV that showed improved adjustment including school attendance, an intervention for families with parental depression that showed improved family coping, and an intervention for youth exposed to war that found reduced depression and trauma outcomes (Rotheram-Borus et al., 2004; Beardslee et al., 2007; Layne et al., 2008). Providing psychoeducation and developmental guidance, developing shared family narratives, enhancing family awareness and understanding, improving family empathy and communication, fostering confidence and hope, supporting effective communication, enhancing family resilience skills (emotion regulation, goal setting, problem-solving, communication, and managing stress reminders), supporting coordinated parent leadership are mechanisms of resilience that FOCUS promotes (Saltzman et al., 2011). Skill-building allows the family to learn the skills in a protected environment and includes application in various situations, including after the treatment is completed. FOCUS for Families is a trauma-informed, eight-module, resilience skill-building family preventive intervention. FOCUS is well-studied and has been shown to improve youth and parent mental health outcomes prosocial behavior in youth, and family functioning for families experiencing significant stressors (Lester et al., 2012, 2016). For Latinx youth in particular, social support and perceived stress influence well-being (Lee et al., 2020). FOCUS for Families was selected for adaptation for delivery to adolescents with depression and their families because of the positive outcomes, including reduction of mental health symptoms, improvement in prosocial behaviors, improved family functioning, and gaining of skills that are helpful in overcoming stress and adversity (Lester et al., 2016).

FOCUS integrates SAMHSA’s (2014) principles of trauma-informed care. FOCUS promotes an environment that is psychologically and physically safe for participants. FOCUS providers establish rapport with the families with whom they are working, set boundaries and expectations about the skill-building approach, and integrate skills that promote safety (e.g., emotion regulation, trauma reminders, goal setting). FOCUS providers are transparent about the model, the skills, and expectations from the provider and the consumer, work to establish trust with families, and acknowledge that the families are the experts on their experiences. FOCUS providers are also collaborative, offer choice, and empower family members. FOCUS recognizes the impact of historical and cultural trauma, discrimination, racism, and bias. Providers integrate the relevant aspects of cultural, historical, and gender issues for each family and provide psychoeducation, methods to manage trauma reminders and additional skill-building as needed.

The present study aims to (1) describe the adaptation of FOCUS for Families for use with adolescents with depression and their families in a school-based clinic setting, resulting in FOCUS for Families with Adolescent Depression (FOCUS-AD); (2) describe the preliminary feasibility and acceptability of FOCUS-AD among adolescents with depression and their families, and (3) present preliminary results of a pilot randomized controlled trial (RCT) comparing FOCUS-AD to usual care in improving depression symptoms and family functioning. We hypothesized that this intervention would be feasible and acceptable as implemented in a school-based setting. We also hypothesized that our results would preliminarily suggest greater improvements in symptoms and family functioning by participants in FOCUS-AD relative to usual care.

## 2. Materials and methods

### 2.1. Community-partnered approach

This study was developed in the context of an academic-community partnership between a large urban school district’s mental health unit and clinician researchers, building on a 20-year collaborative relationship. This academic-community partnership is guided by principles of Community-Partnered Participatory Research (CPPR), with co-planning and consensus between the district clinicians and academic researchers at each phase of the research process (Jones and Wells, 2007). For this study, partnered decision-making included co-designing of the protocols, choice of measures, and implementation and workflow within the school-based mental health clinics. This pilot study was delivered in school-based mental health clinics, during the normal course of care as delivered by district-employed Psychiatric Social Workers (PSWs). The school partners provided valuable input regarding the cultural and social context of the students and families being served. For example, PSWs highlighted the stressors related to immigration and fear of deportation common amongst district families and the common misperceptions and stigma of mental health challenges embedded in the beliefs and attitudes of many in the school district’s communities. Thus, adapting the skills-based FOCUS intervention (Lester et al., 2012, 2016)—which builds on a family’s strengths and has been widely used with culturally diverse families who have experienced trauma—was well-supported by the school partners. In addition to contributing their experience and knowledge working with the communities being served, the school partners played a crucial role in adapting this intervention for implementation within the school-based mental health clinics and in ensuring that the protocol was congruent with their workflow and potentially sustainable. The PSWs considered individual CBT (Chorpita and Weisz, 2009) as their “usual care” for students with depression and suggested that FOCUS-AD be the comparison group.

### 2.2. Setting

The participating school district is a large, urban district that is comprised of students that identify as 74% Latinx, 10% Caucasian/White, and 9% African American/Black; 81% of students qualify for free or reduced-priced meals. Prior research has demonstrated a high burden of stressors among youth across the district (Ramirez et al., 2012), with 19% of high school students screening positive for PTSD in one pilot study (Ijadi-Maghsoodi et al., 2017). The district employs over 450 PSWs, who provide a range of mental health services from primary prevention, targeted prevention, and intensive mental health services. These intensive services, most relevant to the PSW role for this study, include individual and family outpatient therapy in school-based mental health clinics that is both short- and long-term for a variety of mental health challenges, such as depression, anxiety, and disruptive behaviors for district students in grades K-12. In the district’s school-based clinics, for example, PSWs saw 1,515 unique students with over 22,000 encounters in 2016 (personal communication, 2016).

### 2.3. Participants

**Figure 1:**
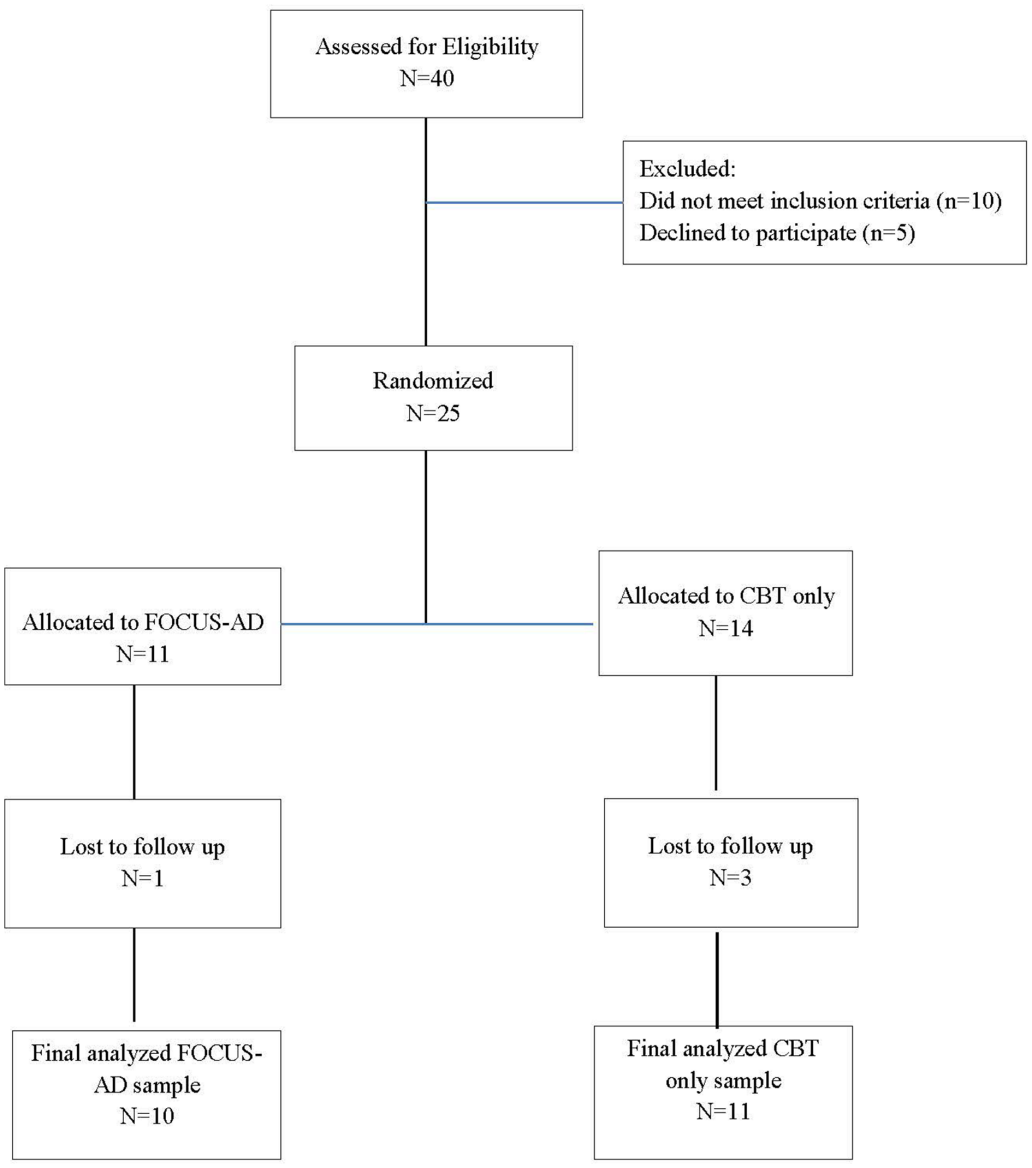
CONSORT diagram: FOCUS-AD vs. CBT only.

Forty students were assessed for eligibility in this study, which included: being 12–18 years old and having a Patient Health Questionnaire (PHQ-8) score of 10 or higher, having at least one parent or caregiver (referred to henceforth as “parent”) who consented to participate in the study with the student assenting, speaking English and/or Spanish (for the parent and student), and being a client at one of the district’s 12 participating school-based mental health clinics. Exclusion criteria resulted in the exclusion of students who were wards of the court or did not have a parent who wanted to participate, students that the school clinician assessed as not having the cognitive or behavioral ability to participate in or benefit from either intervention, students for whom depression was not the primary presenting problem, and students with psychosis or suicidality requiring a higher level of care. Of the 40 students assessed, 25 students and their parent were included in the study (10 did not meet inclusion criteria and 5 declined to participate), with 11 randomly assigned to FOCUS-AD and 14 to CBT only. Of those who were randomized, 21 completed treatment and follow-up assessments (10 in FOCUS-AD and 11 in CBT); with four lost to follow-up (see Figure 1).

### 2.4. Procedures

Students were recruited from 10 district school mental health clinics, ranging from 1 to 9 cases per school-based clinic. As new students received intake evaluations at each participating clinic, clinic staff identified potentially eligible students and provided verbal and written information about the study to the parent and student in their preferred language (English or Spanish). Many families had hesitations about receiving on-going mental health treatment and/or participating in research, particularly given the political climate and the high number of Latinx families served at the clinics. Because this was a community-based and partnered study, the number of families were approached but declined to be assessed was not tracked due to the burden on clinic staff. If families were interested, research staff arranged a time to meet with the parent and student to discuss the study in more detail and administer the PHQ-8 to the student. Students were eligible if they scored a 10 or higher on the PHQ-8, spoke English or Spanish, and both parent and student were interested in participating in the study. Then, the research staff consented parent and assented the student, administered a baseline evaluation, and randomized the family to one of the two interventions (FOCUS-AD or usual care) by referencing a pre-generated randomization list. Follow-up assessments were administered at the end of treatment, approximately 5 months from the initial assessment. The median number of days between baseline and follow-up assessment completion among adolescents was 149 days (IQR = 40.00) and among caregivers was 141 days (IQR = 48.00). Students and parents each received $10 per assessment, for a total of $20 each. This study was approved by the (Institution redacted) IRB and the district’s research review committee. A Certificate of Confidentiality was also obtained from the National Center for Advancing Translational Sciences.

## 3. Interventions

### 3.1. FOCUS-AD

The FOCUS model has been used with multicultural populations of military and non-military families affected by stress and trauma (Saltzman et al., 2008). Given the promising findings of FOCUS for youth and parents (e.g., Lester et al., 2013, 2016), and given the significant burden of trauma and stressors among youth and families in this particular district (Ramirez et al., 2012; Ijadi-Maghsoodi et al., 2017), FOCUS for Families was chosen by the community-academic partnership team to be adapted to treat adolescent depression in this school setting. The core elements of the FOCUS model were maintained and combined with the best practices in CBT treatment for depression. The core elements of FOCUS are: The Family Check-In, Family Narrative, Family Resilience Skills, and Psychoeducation and Developmental Guidance (Beardslee et al., 2013). FOCUS-AD consists of 14 manualized modules, including the entire eight modules of FOCUS for Families and an additional six modules of CBT skills developed specifically to reduce depressive symptoms, combining a family skills-based preventive intervention with CBT (see Table 1). The CBT sessions included specific techniques to target symptoms of adolescent depression. For example, depression-specific psychoeducation is integrated as well as additional skills such as emotional awareness, relaxation strategies, cognitive coping, understanding the relationship between feelings, thoughts, and behaviors, positive self-talk, additional strength-identifying activities, and planning for utilizing coping skills. These sessions integrated skill-building utilizing known CBT tools to reduce depression. All of the study materials were translated into Spanish, including many of the acronyms used in the FOCUS for Families model were interpreted in a culturally responsive manner in order to reinforce the learning of skills. Additionally, all of the PSWs who implemented either treatment in Spanish were bilingual and bicultural. PSWs were familiar with culturally humble approaches to implementing evidence-based practices and have significant experience working with Latinx families.

**Table 1:** Description of FOCUS-AD modules including intended participants and session activities.

Module	Participants	Session activity
1. Introducing parents to FOCUS	Parent(s)/caregiver(s)	Overview of FOCUS Depression education Goal-setting
2. Introducing children to FOCUS	Student and siblings	Introduce FOCUS Introduce emotional awareness Goal-setting
3. Constructing parents’/caregivers’ FOCUS narrative timelines	Parent(s)/caregiver(s)	1. Emotional regulation and communication narrative timeline
4. Emotional awareness	Student	1. Emotional awareness
5. Learning about depression	Student	1. Depression education 2. Emotional awareness
6. Learning to relax	Student and parent(s) /caregiver(s)	1. Calming and grounding activities
7. Cognitive coping	Student	1. Thought distortions 2. Thought swaps
8. Constructing children’s FOCUS narrative maps	Student and siblings	1. Emotional regulation and communication narrative timemap/timeline
9. Preparing parents/caregivers for the family sessions	Parent(s) /caregiver(s)	1. Review narrative timeline 2. Parent prep for family session
10. Developing a FOCUS family narrative	Family	1. Family emotional regulation and communication narrative sharing
11. Building family resilience skills	Family	1. Step-wise problem solving method
12. Presenting a positive self	Student	1. Noticing your strengths
13. Plan for coping and goal-setting	Student	1. Plan for coping 2. Goal-setting
14. Preparing for the future	Family	1. Resilience skills 2. Family ritual or activity 3. Develop ongoing family goals and activities

Fourteen district PSWs were trained in the Families OverComing Under Stress for Families with Adolescent Depression (FOCUS-AD) adaptation by the lead trainer (initials redacted) with at least one PSW from each participating clinic receiving training (one clinic had three trained PSWs). PSWs were trained in a two-day FOCUS for Families training, then were provided with training on the FOCUS-AD intervention, including modules and information on the research study. The FOCUS-AD manual contains fidelity checklists and providers were trained on how to use them to maintain fidelity to the treatment model.

PSWs trained in FOCUS-AD who were assigned cases, received biweekly FOCUS-AD consultation throughout their cases with a lead trainer and through supervision and/or consultation provided by their school clinic. Consultation served as a time to ensure consistency of the model implementation and discuss challenges and successes. Academic partners also worked with district clinicians in determining how the intervention could be flexibly implemented to best serve the students and families while maintaining fidelity. For example, some of the content across two modules was combined into one meeting time at the end of a school year with a family who had missed several appointments. The fidelity checklists were used to ensure all aspects of the intervention were administered with these adaptations.

### 3.2. CBT only

The district PSWs utilize individual CBT (Chorpita and Weisz, 2009) as their usual care intervention, which provides optional parent involvement primarily for psychoeducation regarding depression and skills as needed. All participating clinics had PSWs trained in CBT only as part of their usual care and provided training and consultation as needed by the clinic supervisor.

## 4. Implementation

It was intended that the length of treatment and the number of sessions for both of the interventions was roughly the same (14 sessions), and the interventions were both delivered flexibly to take into account “real-world” implementation issues, such as scheduling challenges.

## 5. Measures

### 5.1. Youth and parent report measures

The assessment concluded with eight questions evaluating adolescent and caregiver satisfaction with the program. These questions constitute the 8-item Client Satisfaction Questionnaire (CSQ) designed to measure satisfaction in health and human service systems (Larsen et al., 1979). For each item, Likert response options are translated to numeric values ranging from 1 to 4 and CSQ Score is calculated by taking the average across all 8 items. CSQ Scores of >3.00 are used to suggest high participant satisfaction.

### 5.2. Youth report measures

The Patient Health Questionnaire-8 (Kroenke and Spitzer, 2002) is an 8-item self-report measure used to diagnose and assess depressive disorders. It is identical to the PHQ-9 (Kroenke et al., 2001, 2009; Razykov et al., 2012), but omits the ninth item that asks about suicidal ideation. Item responses are on a Likert scale and range from 0 = “Not at all” to 3 = “Nearly every day,” with higher scores associated with greater depressive symptoms. Numeric responses to the 8 items are summed to yield a PHQ-8 Total Score. Used to identify clinically meaningful symptoms of depression, a total score of 10 or higher has demonstrated sensitivity and specificity for major depressive disorder of 1.00 and 0.95, respectively, and sensitivity and specificity for any depressive disorder of 0.70 and 0.98, respectively (Kroenke et al., 2009). Adolescent PHQ-8 Total Scores at baseline and follow-up were used in this study with higher scores indicating greater symptomatology. Reliable change was defined as a decrease in PHQ-8 Total Score of ≥5 which has been deemed a clinically significant response to depression treatment (Kroenke and Spitzer, 2002). The clinics had a separate protocol for assessing suicide that they completed for every intake and was not included in this study.

The Child PTSD Symptom Scale (CPSS; Foa et al., 2001) is a self-report scale to assess the symptoms of posttraumatic stress disorder (PTSD), using a 17-item measure of PTSD symptomatology, with item responses on a Likert scale that range from 1 = “Not at all” to 4 = “Always (or 5 or more times a week).” These 17 items are summed to yield a CPSS Total Symptom Severity Score. A psychometric properties study suggests that total symptom severity scores of 16 or higher can be used to identify clinically meaningful symptoms of PTSD while establishing an optimal balance between sensitivity and specificity (Nixon et al., 2013). Adolescent CPSS Total Symptom Severity Scores at baseline and follow-up were used in this study with higher scores indicating greater symptomatology. Reliable change was defined as a decrease in CPSS Total Symptom Severity Score of ≥8.98 based on calculation of the reliable change index with assumed test–retest reliability of 0.84 and standard deviation of 8.1 (Foa et al., 2001).

The Strength and Difficulties Questionnaire—Child Report (SDQ; Goodman et al., 1998) is a brief 25-item self-report measure that assesses positive and negative behavioral attributes. The child self-report version can be completed by those aged 11–16 years. The SDQ results in 4 subscales measuring negative behavioral attributes (conduct problems, inattention-hyperactivity, emotional symptoms, and peer problems). Item responses are measured on a Likert scale with range from 0 = “Not True” to 2 = “Certainly True.” Used as an overall summary measure, a SDQ Total Difficulties Score can be obtained by summing items across all 4 subscales. A total difficulties score of 16 or higher is used to identify high difficulties (Goodman et al., 2003). Adolescent SDQ Total Difficulties Scores at baseline and follow-up were used in this study with higher scores indicating more difficulties. Reliable change was defined as a decrease in SDQ Total Difficulties Score of ≥8.36 based on prior literature (Wolpert et al., 2014).

Adolescent coping was provided through the Brief COPE (Carver, 1997) a 28-item measure designed to help identify and assess coping and actions, developed as a brief-form of the longer COPE inventory (Carver et al., 1989). It is comprised of 14 subscales, including: self-distraction, active coping, denial, substance use, use of emotional support, use of instrumental support, behavioral disengagement, venting, positive reframing, acceptance, planning, humor, religion, and self-blame, though we did not assess use of instrumental support or self-blame. Item responses are on a Likert scale and range from 0 = “I did not do this at all” to 3 = “I did this a lot.” Item responses are on a Likert scale and range from 1 = “I did not do this at all” to 4 = “I did this a lot.” Each subscale consists of two items and subscale scores thus range from 2 to 8. Scores of 5 or higher were assumed to indicate use of the coping mechanism.

### 5.3. Parent-report measures

The McMaster Family Assessment Device (FAD; Epstein et al., 1983) is a 60-item measure that assesses various characteristics of families and family functioning. Item responses are on a Likert scale and range from 1 = “Strongly Agree” to 4 = “Strongly Disagree,” with higher scores associated with more problematic functioning. Included within the FAD is a 12-item General Functioning subscale that provides an overall measure of family adjustment (Byles et al., 1988). The FAD General Functioning Score is calculated by summing numeric responses across all 12 items. A score of 2.0 or higher is used to identify unhealthy family functioning and is associated with a sensitivity of 0.67 and specificity of 0.64 (Miller et al., 1985). Parent-reported FAD General Functioning Scores at baseline and follow-up were used in this study with higher scores indicating less healthy family functioning. Reliable change was defined as a decrease in FAD General Functioning Score of ≥0.67 based on calculation of the reliable change index with assumed test–retest reliability of 0.71 and standard deviation of 0.45 (Miller et al., 1985).

### 5.4. Participant characteristics

In addition to the above measures, participants were asked a series of demographic questions at baseline, including race, ethnicity, age, and gender for both adolescents and caregivers, as well as marital and employment status for caregivers. We evaluated adolescents’ use of mental health services through three yes/no questions, asked of the caregiver, to help identify sources of professional help for any emotional or behavioral problems.

Parent participants also provided information about their own mental health symptoms, which were described at baseline for this sample. Parents completed the previously described PHQ-8 and the PTSD Checklist—Civilian Version (PCL-C; Weathers et al., 1993), to measure depression and PTSD symptoms, respectively. The PCL-C is comprised of 17 items designed to assess the primary symptoms of PTSD associated with a traumatic event as outlined by the *DSM-IV*. Item responses are on a Likert scale and range from 1 = “Not at all” to 5 = “Extremely,” with higher scores indicating higher stress and PTSD symptomatology. A PCL Total Symptom Severity score is obtained by summing over all 17 items, with scores of 30 or higher indicating clinically meaningful symptoms of PTSD with a sensitivity of 0.78 and specificity of 0.88 (Bliese et al., 2008).

## 6. Statistical analyses

To describe the sample of participating adolescents and caregivers at baseline, frequencies, percentages, medians and interquartile ranges were calculated among FOCUS-AD and CBT only arms. Statistical analyses were conducted using SAS Version 9.4. To inform acceptability, frequencies and percentages of adolescents and caregivers providing positive responses to each of the CSQ items were calculated within arms and compared across arms using Fisher’s Exact Tests. For our purposes, a positive response refers to either of the 2 response options denoting the highest levels of satisfaction. Nonparametric tests referenced previously were used to compare CSQ Scores between arms and to compare percentages of participants with CSQ Scores >3.00.

To compare changes in adolescent mental health and family functioning between adolescents randomized to the FOCUS-AD versus CBT only arm, we calculated mean and median changes from baseline to follow-up on the following measures: Adolescent-reported PHQ-8 Total Score, SDQ Total Difficulties Score, and CPSS Total Symptom Severity Score, and parent-reported FAD General Functioning. Due to the small sample sizes, nonparametric tests relying on fewer distributional assumptions were used. Wilcoxon Signed Rank Tests were used to assess for significant changes from baseline to follow-up within each arm. To evaluate preliminary efficacy, Wilcoxon Rank-Sum Tests were used to assess for significant differences in these changes between arms. Frequencies and percentages of adolescents and parents demonstrating reliable change from baseline to follow-up on each measure were calculated.

## 7. Results

### 7.1. Characteristics of youth and caregivers at baseline

Most adolescents participating in the study self-identified as female (81%) and Latinx (86%, see Table 2). Median age among adolescents was 14 years [interquartile range (IQR) = 2.00]. A relatively high percentage reported their emotional/mental health as being fair or poor (86%). From youth-report at baseline, 95% percent of adolescents met the criteria for high difficulties on the SDQ Total Difficulties scale and 100% met the criteria for clinically meaningful PTSD symptoms. In terms of coping strategies, youth most commonly reported using self-distraction (86%) and behavioral disengagement (76%). Based on parent-report of service use, 38% of adolescents had received prior outpatient mental health services from a community mental health clinic, mental health counselor, physician, or day program, and 29% had received services from a hospital, treatment center, group or foster home, juvenile justice facility, or emergency shelter.

**Table 2:** Participant characteristics at baseline among families randomized to the FOCUS-AD and CBT only groups.

	FOCUS-AD (*N* = 10)	CBT only (*N* = 11)	Overall (*N* = 21)
**Adolescent characteristics**	***n* (%)**		
Gender			
Male	2 (20.00)	2 (18.18)	4 (19.05)
Female	8 (80.00)	9 (81.82)	17 (80.95)
Age			
Years, median (IQR)	14.5 (2.00)	13 (3.00)	14 (2.00)
Race/ethnicity			
African American	–	1 (9.09)	1 (4.76)
Caucasian	1 (10.00)	–	1 (4.76)
Latino	9 (90.00)	9 (81.82)	18 (85.71)
Other	–	1 (9.09)	1 (4.76)
Time, baseline to follow-up			
Days, median (IQR)	149 (88.00)	145 (38.00)	149 (40.00)
In general, would you say your emotional/mental health is…			
Excellent/very good/good	2 (20.00)	1 (9.09)	3 (14.29)
Fair/poor	8 (80.00)	10 (90.91)	18 (85.71)
Child received professional mental health services from:^a^			
*Hospital, treatment center, group or foster home, juvenile justice facility or emergency shelter?*			
Yes	2 (20.00)	4 (36.36)	6 (28.57)
*Community mental health clinic, private counselor’s office, physician’s office, or day program?*			
Yes	3 (30.00)	5 (45.45)	8 (38.10)
SDQ: total difficulties			
High difficulties^b^	10 (100.00)	10 (90.91)	20 (95.24)
PTSD			
Clinically meaningful^c^	10 (100.00)	11 (100.00)	21 (100.00)
Brief COPE^d^ coping (clinically meaningful)			
Self-distraction	9 (90.00)	9 (81.82)	18 (85.71)
Active coping	5 (50.00)	4 (36.36)	9 (42.86)
Denial	7 (70.00)	2 (18.18)	9 (42.86)
Substance use	2 (20.00)	1 (9.09)	3 (14.29)
Emotional support	5 (50.00)	7 (63.64)	12 (57.14)
Behavioral disengagement	8 (80.00)	8 (72.73)	16 (76.19)
Venting	7 (70.00)	5 (45.45)	12 (57.14)
Positive reframing	6 (60.00)	2 (18.18)	8 (38.10)
Planning	7 (70.00)	4 (36.36)	11 (52.38)
Humor	4 (40.00)	2 (18.18)	6 (28.57)
Acceptance	6 (60.00)	5 (45.45)	11 (52.38)
Religion	3 (30.00)	1 (9.09)	4 (19.05)
**Caregiver characteristics**	***n* (%)**		
Gender			
Male	1 (10.00)	2 (18.18)	3 (14.29)
Female	9 (90.00)	9 (81.82)	18 (85.71)
Age			
Years, median (IQR)	43 (15.00)	40 (13.00)	41 (11.00)
Race/ethnicity			
African American	–	1 (9.09)	1 (4.76)
Caucasian	1 (10.00)	–	1 (4.76)
Latino	9 (90.00)	10 (90.91)	19 (90.48)
Other	–	–	–
Marital status			
Married/cohabitating	5 (50.00)	5 (45.45)	10 (47.62)
Other^e^	5 (50.00)	6 (54.55)	11 (52.38)
Employment			
Full time or part time	4 (40.00)	6 (54.55)	10 (47.62)
Not currently working	6 (60.00)	5 (45.45)	11 (52.38)
Education			
Did not finish high school	6 (60.00)	6 (54.55)	12 (60.00)
High school and above	3 (30.00)	5 (45.45)	8 (40.00)
Depression			
Clinically meaningful^f^	4 (40.00)	2 (18.18)	6 (28.57)
PTSD			
Clinically meaningful^g^	5 (50.00)	4 (36.36)	9 (42.86)
Time, baseline to follow-up			
Days, median (IQR)	151 (80.00)	140 (44.00)	141 (48.00)
**Family characteristics**	***n* (%)**		
General functioning^a^			
Unhealthy family functioning^h^	7 (70.00)	6 (54.55)	13 (61.90)

a Reported by the caregiver.

b SDQ total difficulties score ≥ 16.

c CPSS score ≥ 16 (17-item version).

d For each coping scale, a score ≥ 5 indicated use of the coping mechanism.

e Other includes: single, divorced, separated, and widowed.

f PHQ-8 total score ≥ 10.

g PCL-C score ≥ 30.

h FAD general functioning score ≥ 2.0.

The vast majority of parent participants were female (86%) and Latinx (90%). Twenty-nine percent of caregivers met the criteria for clinically meaningful symptoms of depression at baseline and 43% met the criteria for clinically meaningful symptoms of PTSD. In reporting on their families at baseline, 62% of parents indicated unhealthy family functioning. All 21 participating adolescents and 29% of parents completed the surveys in English, with the remaining parents participating in Spanish. No significant differences in participant characteristics were found between participants in the FOCUS-AD and the CBT only groups (see Table 2 for details).

### 7.2. Comparisons between baseline and follow-up in FOCUS-AD and CBT only groups

To evaluate the primary outcome of preliminary feasibility and acceptability of the FOCUS-AD and CBT only interventions among adolescents with depression and their families in a school-based setting, we assessed satisfaction and dropout rates. Mean CSQ scores among adolescents or parents in the FOCUS-AD and CBT only groups did not differ significantly [FOCUS-AD Mean (M) = 3.18, CBT only M = 3.50, *p* = 0.15; FOCUS-AD M = 3.64, CBT only M = 3.53, *p* = 0.67; see Table 3]. The mean scores for both treatments indicate that adolescents and parents in both treatment groups were highly satisfied. Of the 25 families who were randomized, 4 dropped out, 1 from the FOCUS-AD group and 3 from the CBT only group. The average time from baseline to follow-up was 151 days for FOCUS-AD and 140 days for CBT only for caregivers and 149 days for FOCUS-AD and 145 days for CBT only for adolescents.

**Table 3:** Percentage of participants in the FOCUS-AD and control arms endorsing each of the following items from the Client Satisfaction Questionnaire (CSQ) at follow-up.

	FOCUS-AD		CBT only	
	*N* (%)		*N* (%)	
	Adolescent (*N* = 10)	Caregiver (*N* = 10)	Adolescent (*N* = 11)	Caregiver (*N* = 11)
How would you rate the quality of service you have received?^a^	10 (100.00)	10 (100.00)	10 (90.91)	11 (100.00)
Did you get the kind of service you wanted?^b^	8 (80.00)	10 (100.00)	11 (100.00)	11 (100.00)
To what extend has our program met your needs?^c^	6 (60.00)	9 (90.00)	9 (81.82)	11 (100.00)
If a friend were in need to similar help, would you recommend our program to him/her?^d^	9 (90.00)	10 (100.00)	11 (100.00)	11 (100.00)
How satisfied are you with the amount of help you have received?^e^	10 (100.00)	10 (100.00)	10 (90.91)	11 (100.00)
Have the services you received helped you to deal more effectively with your problems?^f^	10 (100.00)	10 (100.00)	11 (100.00)	11 (100.00)
In an overall, general sense, how satisfied are you with the service you have received?^e^	8 (80.00)	10 (100.00)	11 (100.00)	11 (100.00)
If you were to seek help again, would you come back to our program?^d^	7 (70.00)	10 (100.00)	11 (100.00)	11 (100.00)
CSQ Score, Mean (SD)	3.18 (0.52)	3.64 (0.36)	3.50 (0.39)	3.53 (0.40)
CSQ Score > 3.00, *N* (%)	5 (50.0)	9 (90.0)	9 (81.8)	9 (81.8)

a % responding “Good” or “Excellent.”

b % responding “Yes, generally” or “Yes, definitely.”

c % responding “Most of my needs have been met” or “Almost all of my needs have been met.”

d % responding “Yes, I think so” or “Yes, definitely.”

e % responding “Mostly satisfied” or “Very satisfied.”

f % responding “Yes, they helped” or “Yes, they helped a great deal.”

Adolescent depression symptoms decreased significantly from baseline to follow-up within the two groups [FOCUS-AD median decrease (MD) = 10, *p* = 0.02; CBT only MD = 6, *p* = 0.01; see Table 4]. Similarly, significant decreases within treatment groups from baseline to follow-up were seen for SDQ Total Difficulties (FOCUS-AD MD = 6.5, *p* = 0.002; CBT only MD = 4.5, *p* = 0.03) and PTSD symptoms (FOCUS-AD MD = 12.5, *p* = 0.01; CBT only MD = 7, *p* = 0.04). However, these symptom decreases did not differ significantly across the two groups. Parent-reported improvements in family functioning were not significant and did not differ between the two groups (FOCUS-AD MD = 0.17; CBT only MD = 0.08).

**Table 4:** Comparisons between baseline to follow-up in adolescent mental health and family functioning in the FOCUS-AD and CBT only groups.

	FOCUS-AD			Wilcoxon signed-rank test	RC	CBT only			Wilcoxon signed-rank test	RC	Wilcoxon rank-sum test
	*N*	Mean (SD)	Median (IQR)	*p*-value	*n* (%)	*N*	Mean (SD)	Median (IQR)	*p*-value	*n* (%)	*p*-value
**Adolescent mental health**											
Δ PHQ-8 total score	10	7.8 (6.83)	10.00 (8.00)	0.02	6 (60.0)	11	5.09 (4.74)	6.00 (8.00)	0.01	6 (54.6)	0.27
Δ SDQ total difficulties score	10	6.30 (2.95)	6.50 (4.00)	0.002	2 (20.0)	10	4.10 (4.58)	4.50 (8.00)	0.03	2 (18.2)	0.34
Δ CPSS total symptom severity score	10	12.90 (12.19)	12.50 (17.00)	0.01	6 (60.0)	11	9.27 (12.93)	7.00 (17.00)	0.04	4 (36.4)	0.60
**Family functioning** ^a^											
Δ FAD general functioning score	9	0.11 (0.24)	0.17 (0.25)	0.27	0 (0.0)	10	0.09 (0.25)	0.08 (0.33)	0.33	0 (0.0)	1.00

a Reported by the caregiver.

RC, reliable change.

## 8. Discussion

The findings from this pilot study suggest that the FOCUS-AD intervention appears to be both feasible and acceptable, as delivered in a school-based clinic setting with this predominantly Latinx adolescent population. Once in treatment, students and their families had a high retention rate in treatment. Those who received FOCUS-AD also generally reported that the intervention was acceptable, with reasonable satisfaction reported by parents and students. Satisfaction was slightly higher for caregivers than students for the FOCUS-AD intervention, which may reflect the developmentally appropriate desire for adolescents to individuate from their caregivers.

Our baseline findings highlight a need for services for those adolescents with depression presenting to school-based mental health clinics. Given that 50% of children and adolescents will have mental health challenges, school-based clinics are one of the ways to provide accessible services (Committee on School Health, 2004; Merikangas et al., 2010; SAMHSA, 2022); and previous studies have shown that Latinx populations are less likely to access mental health services outside of the school (Kataoka et al., 2007). These findings indicate that a family-centered approach could help enhance family involvement in treatment, especially in under-resourced communities where structural barriers to care are high.

In addition, we explored how FOCUS-AD, a combination of a family-based intervention with individual CBT, compared to CBT only, in improving mental health symptoms. This study suggests that both treatments delivered in school-based health clinics, may be helpful. Given the small sample size, we are tentative in our conclusions. However, the findings of this small pilot are helpful in determining that additional research in this area would be supportive to families with adolescents with depression.

As seen in other studies in the literature (Vibhakar et al., 2019), the adolescents in our study not only had clinically significant depressive symptoms at baseline but also universally reported posttraumatic stress symptoms in the clinical range. We found that FOCUS-AD and the CBT only treatments significantly reduced adolescent mental health symptoms, including depression and trauma symptoms, as well as general emotional and behavioral problems. However, no difference between treatment groups was found. Treatment of moderate and severe adolescent depression is challenging and studies often find no or low effect size (Thapar et al., 2012). A larger school-based RCT study of Chilean adolescents with depression did not find a significant reduction of depression symptoms comparing a school-based CBT intervention to a control group receiving no interventions (Gaete et al., 2016). Additionally, a recent meta-analysis of school-based interventions for adolescent depression found that these treatments had a small effect on reducing depression symptoms, which is not dissimilar from adolescent depression treatment in other settings (Gee et al., 2020). Further, research in this area is not vast, with most interventions focusing on the prevention of depression, rather than treatment (Bevan Jones et al., 2018).

Families are seldom included in research studies for adolescent depression, despite family inclusion being considered optimal for depression treatment (Tursi et al., 2013), including in school-based settings (Bevan Jones et al., 2018; Gee et al., 2020). Somewhat surprisingly, in the present study, neither intervention significantly improved family functioning. Family functioning plays an important role in adolescent mental health. For example, research demonstrates that parental closeness and family functioning are associated with lower levels of depression among Latinx youth (Perreira et al., 2019). Others have described the positive role that family cohesion and support can play, in particular, for Latinx youth in preventing depression (Potochnick and Perreira, 2010; Perez et al., 2011). In the FOCUS-AD group, approximately 40% of the sample started with what is considered “healthy” family functioning, which slightly improved although not significantly. Future research should replicate this pilot with a larger sample size and longer follow-up period to determine if family functioning improves with FOCUS-AD.

The parents and families in this study were highly distressed, which was not an inclusion requirement. This is not surprising given that a family history of depression is a risk factor for adolescent depression (Thapar et al., 2012). There are other compelling reasons to include caregivers in interventions. A study found that Latinx teenagers with parents who had greater knowledge about depression were more likely to seek treatment for depression (Chandra et al., 2009). Adolescents who experience greater depression tend to receive less social support from parents/caregivers (Piña-Watson and Castillo, 2015). These findings further emphasize the importance of family involvement in adolescent depression treatment.

Coping skills are some of the few changeable risk factors for depression and often an integral part of depression treatment. Participants in this study reported potentially maladaptive coping strategies such as self distraction and behavioral disengagement on the Brief COPE. Although it was beyond the scope of this pilot to evaluate the mechanism(s) of change in coping approaches as a result of treatment, our preliminary findings suggest that addressing maladaptive coping strategies identified at baseline could be important to target during treatment. Increased behavioral disengagement and distraction could be indicative of a general avoidance of stressors. The understanding of baseline coping strategies could be used to tailor the intervention, build on youths’ existing strengths, and reduce maladaptive coping, which may promote well-being and lower stress (García and Wlodarczyk, 2018).

Our findings did not support our secondary hypothesis that FOCUS-AD would be superior to the CBT only treatment in reducing symptoms and improving family functioning; it does provide results indicating that FOCUS-AD is a promising intervention and further study is needed. Although we found promising results, there are several limitations of this pilot study. First, we had a small sample size, which limited our ability to reasonably compare the two interventions adequately and to interpret and generalize statistically significant results. PSWs also reported challenges in referring parents to a family intervention which would necessitate missing work. School settings—in which parents may face barriers to attending school clinics—may not be ideal for this particular family-based intervention, however, given the recent advances in the availability of telehealth during the COVID-19 pandemic (e.g., Kodjebacheva et al., 2023), involving families in remote care that is initiated through schools is an area for future study. Also of note, this study was completed prior to the COVID-19 pandemic and before telehealth was offered in the school district. Preliminary findings supporting telehealth family interventions, including FOCUS for military-involved families, are promising (Mogil et al., 2022). Future studies would also benefit from a mixed methods approach with both quantitative and qualitative feedback from providers, students, and caregivers, to provide a contextual understanding of implementing the intervention among youth and families.

Given the limitations of conducting the pilot within busy school mental health clinics and the burden on clinical staff, we did not systematically collect information on how many sessions were completed nor on fidelity to the intervention, nor do we know the reasons for refusal of the intervention or drop-out. We do know that relatively few families dropped out of the study once they started. Fidelity checklists are available for each FOCUS-AD module and PSWs were trained on using the fidelity checklists, and fidelity was addressed during consultation calls. The checklists were not systematically collected as a part of the study. It is possible that because families needed to be available during school hours to participate in the study that there was more family involvement in the CBT intervention than typical for this setting. We struggled with recruitment of youth and families. Mufson et al. (2004) outline the multiple challenges in conducting research in school-based clinics such as youth reluctance to include their families in treatment, the burden of completing measures for the research study, challenges with the randomization process, and the time and resources needed to train clinicians to participate in the study. Several of these factors may have played a role in our recruitment as well. García et al. (2017) discuss various reasons why Latinx individuals may be hesitant to participate in research, such as distrust, fear of discrimination, concerns about confidentiality, and a lack of understanding about the research process. To reduce stigma, we did not collect information on documentation status, however, it is likely that at least some of the participants were undocumented or had undocumented family members. Undocumented Latinx individuals report lower access to mental health services than documented US-born Latinx individuals (Ortega et al., 2018) and potential participants who were undocumented immigrants may not have participated because they viewed treatment as futile as mental health services do not address immigration-related stress and/or participants may have a lower perceived need because of the normalization of their stress (Cha et al., 2019).

While our study had a smaller-than-anticipated sample size, a small sample size is justified for a small pilot randomized trial (Whitehead et al., 2016), such as the present study. Some studies even recommend a small sample size at or close to that of the present study (e.g., Kieser and Wassmer, 1996; Julious, 2005). This present study did not aim to obtain an ideal power of 0.8 or above, which would have required approximately 75 families in each of the two arms of the study (based on the observed PHQ outcomes), as this is beyond the scope and resources of this pilot. Additionally, we encountered difficulties in recruitment, which is a valuable lesson learned about challenges with recruitment and retention with this population. The data we gathered does inform potential future effectiveness trials and calculations, a key outcome of pilot studies (Moore et al., 2011).

Further research in this area is warranted as the FOCUS for Families model has been shown to improve mental health symptoms for children and caregivers experiencing stressors in large-scale evaluations (Lester et al., 2016). The best practice in adolescent depression treatment includes families; the FOCUS-AD model decreases barriers for clinicians to be comfortable integrating families and enhances their skills in their work with youth. FOCUS-AD may require more effort on the part of the school clinician to coordinate with parents’ schedules and bring in families to the sessions rather than providing individualized CBT with only the adolescent and clinicians must participate in a two-day training in the model. However, we developed the intervention given that our partnered schools wished to offer an intervention that more fully engages parents/caregivers to address the stressors experienced by the family system because of the research outcomes when families are involved in adolescent depression treatment (Tursi et al., 2013; Reyes-Portillo et al., 2017; Bevan Jones et al., 2018; Gee et al., 2020), and because previous studies have shown reduction of mental health symptoms across the family unit (e.g., Lester et al., 2016). Combining this approach with CBT is less burdensome for the providers than having to offer separate CBT and family therapy sessions. The other advantage is that, unlike most individual CBT or family therapy, this one was adapted for delivery in the school setting which improves overall access to care and minimizes some barriers. For schools that wish to take a family-focused approach and that have the desire to engage more with parents, FOCUS-AD is a potentially promising intervention to use.

## 9. Conclusion

Schools are an important place for providing mental health services, in addition to education. It appears that school-based interventions that focus on prevention and early intervention of depression may be effective (Calear and Christensen, 2010) and there is a need for effective interventions for depression. Given that up to 50% of adolescents have experienced a mental health disorder at some point (Merikangas et al., 2010; SAMHSA, 2022) and previous studies have shown that Latinx populations are less likely to access mental health services outside of the school (Kataoka et al., 2007). Our findings indicate that a family-centered approach could help enhance family involvement in treatment, especially in under-resourced communities where structural barriers to care are high. Given the goals of schools to enhance parent and family engagement, and barriers that we noted in our study, schools may consider offering family-based services that are beyond school hours, held in alternative locations, or through telehealth to increase accessibility, especially for parents, and make efforts to reduce stigma and challenges in accessing services. This study supports the need for further investigation of family involvement in treatment. It adds to the small body of limited research indicating that skills-based interventions are promising for the treatment of adolescent depression among minoritized and under-resourced youth and families. Further studies with a larger sample size are needed to make more conclusive statements about the treatment approach.

This study found that predominately Latinx adolescent participants and their caregivers seeking care at a school mental health clinic experienced significant distress. Both FOCUS-AD and CBT were effective in reducing depression and PTSD symptoms. There were no changes in family functioning for either intervention, although both interventions were satisfactory to the families receiving treatment. A skills-based family intervention which has previously been used with families who are highly stressed and experienced trauma appears to be a promising model for treating adolescent depression in schools and reaching family members who have also been affected by life stressors (Lester et al., 2016).

## Data availability statement

The datasets presented in this article are not readily available because they were accessed with a Data Use Agreement that restricts data sharing outside this project. Requests to access the datasets should be directed to Dr. Marlotte.

## Ethics statement

The studies involving humans were approved by University of California Los Angeles. The studies were conducted in accordance with the local legislation and institutional requirements. Written informed consent for participation in this study was provided by the participants’ legal guardians/next of kin.

## Author contributions

SK, LM, and PL contributed to the conception and design of the study. HA and AK organized the database. AK and HA performed the statistical analysis. LM wrote the first draft of the manuscript. SK, RI-M, KG, AK, and HA wrote sections of the manuscript. All authors contributed to the article and approved the submitted version.

## Funding

Research was supported by the UCLA Clinical and Translational Science Institute (NIH NCATS UCLA CTSI Grant number UL1TR000124) and the UCLA Children’s Discovery and Innovation Institute (CDI). During the preparation of this manuscript, RI-M received funding from the National Institute on Drug Abuse of the National Institutes of Health under Award Number K12DA000357. SK, RI-M, and HA are funded by National Institute of Mental Health (1U01MH131827-01) and consult to the Los Angeles Unified School District.

## Conflict of interest

The authors declare that the research was conducted in the absence of any commercial or financial relationships that could be construed as a potential conflict of interest.

## Publisher’s note

All claims expressed in this article are solely those of the authors and do not necessarily represent those of their affiliated organizations, or those of the publisher, the editors and the reviewers. Any product that may be evaluated in this article, or claim that may be made by its manufacturer, is not guaranteed or endorsed by the publisher.
